# The Mechanism of a Retro-Diels–Alder Fragmentation of Luteolin: Theoretical Studies Supported by Electrospray Ionization Tandem Mass Spectrometry Results

**DOI:** 10.3390/molecules27031032

**Published:** 2022-02-03

**Authors:** Magdalena Śliwka-Kaszyńska, Iwona Anusiewicz, Piotr Skurski

**Affiliations:** 1 Department of Organic Chemistry, Faculty of Chemistry, Gdańsk University of Technology, Narutowicza 11/12, 80-233 Gdańsk, Poland; magdalena.sliwka-kaszynska@pg.edu.pl; 2 Laboratory of Quantum Chemistry, Faculty of Chemistry, University of Gdańsk, Wita Stwosza 63, 80-308 Gdańsk, Poland; iwona.anusiewicz@ug.edu.pl

**Keywords:** flavonoids, luteolin, fragmentation mechanism, retrocyclization, liquid chromatography mass spectrometry

## Abstract

The mechanisms of retro-Diels–Alder fragmentation of luteolin are studied theoretically using the Density Functional Theory method (B3LYP hybrid functional) together with the 6-311++G(d,p) basis set and supported by electrospray ionization tandem mass spectrometry (ESI-MS) results. The reaction paths leading to the formation of ^1,3^A^−^ and ^1,3^B^−^ fragment ions observed as the main spectral features in the ESI-MS spectrum are described and discussed, including the structures of the transition states and intermediate products. The heights of the activation energy barriers which have to be overcome along the reaction paths corresponding to 1,3-retrocyclization cleavage of the ionized luteolin are predicted to span the 69–94 kcal/mol range (depending on the initial isomeric structure) for the concerted retrocyclization mechanism and the 60–89 kcal/mol (first barrier) and 24–52 kcal/mol (second barrier) barriers for the stepwise mechanism (also depending on the initial isomeric structure). It is also demonstrated that the final fragmentation products (^1,3^A^−^ and ^1,3^B^−^) are in fact represented by various isomeric systems which are not experimentally distinguishable. In addition, the absence of the spectral feature corresponding to the [M-B]^−^ fragment ion formed by the rupture of the C-C bond connecting luteolin’s B and C rings (which does not occur during the ESI-MS experiment) is explained by much larger energy barriers predicted for such a process.

## 1. Introduction

Flavonoids are natural polyphenol compounds widely distributed in the plant kingdom. They abundantly occur in almost all parts of plants including roots, heartwoods, sapwoods, barks, foliage, fruits, and flowers [1,2]. Apart from being the most common yellow natural colorants, flavonoids exhibit a wide range of biological activities [3,4,5,6]. As such bioactive compounds, these systems are of great interest in nutrition and pharmacology, mostly due to their outstanding anti-inflammatory, antioxidant, antibacterial, antifungal, and antitumor properties [2,7,8,9,10]. Among numerous flavonoids, luteolin is a substance found in many vegetables, e.g., celery, broccoli, green pepper, sweet bell peppers, carrots, onion leaves, parsley, and thyme [11]. As demonstrated in several studies, luteolin possesses anti-inflammation, anti-allergic, and anticancer activities [12,13,14]. Therefore, efforts to improve both the detection and identification of luteolin in various biological matrices are being continuously undertaken.

**Figure 1:**
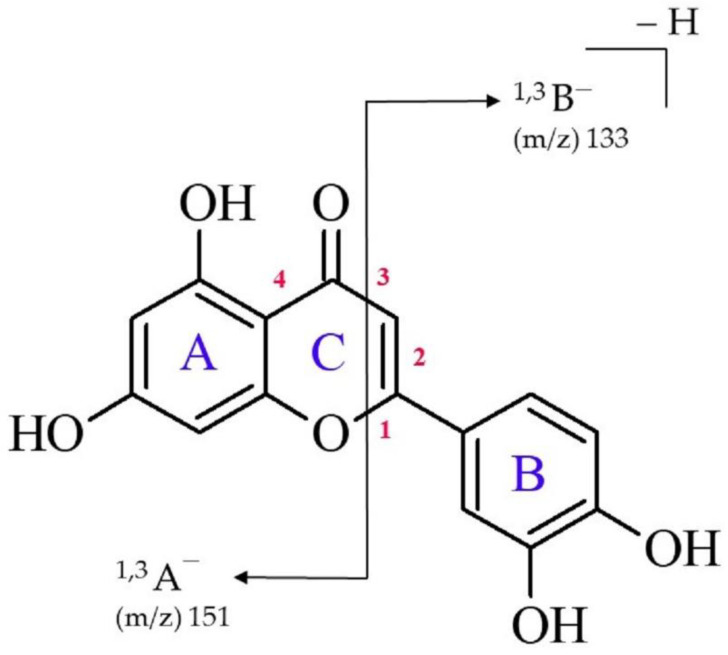
Chemical structure of luteolin with the numeration of bonds within the **C** ring and the indication of retrocyclization cleavages (RDA fragmentation) observed in this study.

From the chemical point of view, luteolin (2-(3,4-dihydroxyphenyl)-5,7-dihydroxy-4H-chromen-4-one, see Figure 1) is a hydroxylated phenolic molecule composed of a skeleton containing two benzene rings (labeled **A** and **B**) connected via a heterocyclic γ-pyrone ring (labeled **C**). High-performance liquid chromatography (HPLC) coupled to electrospray ionization tandem mass spectrometry (ESI-MS) are convenient techniques which enable the separation and identification of individual flavonoids [15,16,17,18,19]. Due to the great number of flavonoids commonly existing in plant matrices, it is important to investigate the fragmentation pathways of a given substance to obtain appropriate information regarding its structure. The main fragment pathway of flavonoids within aglycone is the retro-Diels–Alder (RDA) reaction coupled to losses of small neutral molecules and fragments, e.g., CO_2_, CO, H_2_O, and C_3_O_2_ [20]. In general, the most useful RDA fragmentation of luteolin involves the cleavage of two bonds in the **C** ring (labeled 1 and 3 in Figure 1), leading to the formation of ^1,3^A^−^ and ^1,3^B^−^-negative ions which provides information on the number and nature of substituents in the **A** and **B** rings [18,20,21]. These fragmentation peaks contribute to the mass spectral fingerprint, and the ^1,3^A^−^ often represents the major fragment ion of flavones in the negative ion mode. According to several prior studies, the structure of a ^1,3^A^−^ fragment in positive ion mode (ESI+) is suggested to have a ketene-like structure, whereas in negative ion mode, it corresponds to a lactone [15,17,22,23].

Albeit the possible mechanism of retrocyclization cleavage leading to the formation of the ^1,3^A^−^ and ^1,3^B^−^ ions in the case of flavones was proposed in the course of the earlier reports; it was only based on the speculations derived from the arrow-pushing (i.e., electron-pushing) technique. Clearly, the use of such a formalism only provides a vague idea of the progression of the reaction mechanism (as the electron density does not move around so discretely in reality) rather than an insight into the process at a molecular level. Therefore, other research tools (either experimental or theoretical) are required to build the reliable conjecture which describes in detail what takes place at each stage of an overall RDA reaction involving luteolin.

In this work, we present the results of our negative ion mode (ESI-)-MS study followed by theoretical findings concerning the concerted and stepwise reaction mechanisms which lead to ^1,3^A^−^ and ^1,3^B^−^ anions formed via the 1,3-retrocyclization cleavage of the ionized luteolin molecule, and then, we move on to characterize (on the basis of our theoretical predictions) an alternative fragmentation process (i.e., the rupture of the C-C bond connecting **B** and **C** rings) which does not occur during the ESI-MS experiment.

## 2. Materials and Methods

### 2.1. Chemicals and Materials

Raw dyestuff material from weld (*Reseda luteola* L.) was obtained from Kremer Pigmente (Aichstetten, Germany) in dried form and was homogenized prior to the extraction process. Acetonitrile and methanol used as mobile phase components (HPLC grade) were purchased from Merck (Darmstadt, Germany). Hydrofluoric acid (98–100%) was purchased from Fisher Scientific (Hampton, NH, USA). Dimethyl sulfoxide (DMSO, ACS grade) was obtained from Merck KGaA (Darmstadt, Germany).

### 2.2. Equipment

Chromatographic analysis was performed using Agilent liquid chromatograph series 1290 (Agilent Technology, Waldbronn, Germany) consisting of binary pump G4220A, autosampler G4226A, thermostated column compartment G1316C, diode-array detector G1315C, and triple quadrupole mass spectrometer G6460 with AJS electrospray ionization source. The chromatographic system was controlled with Agilent MassHunter software B 06.01.

### 2.3. Extraction of Dyes from Weld

The plant raw material used as the source of a number of flavonoids, including luteolin, was extracted according to the HF procedure [24,25,26,27]. Briefly, dyestuffs were extracted from the homogenized plant material (estimated weight: 10 mg) in an ultrasonic bath for 0.5 h (2 × 15 min) at a temperature not exceeding 40 °C, using 500 μL of the mixture containing 8 M hydrofluoric acid/MeOH/ACN/DMSO (2:1:1:1, *v*/*v*). The mixture was centrifuged at 9000 rpm for 5 min to separate the particulate matter. The supernatant was filtered over a 0.45 μm RC syringe filter. The concentration of luteolin in the extract was estimated as 5 μg/mL.

### 2.4. LC-MS Analysis

The samples (2 μL) were injected onto a Poroshell EC-C18 2.7 µm (3.0 mm × 150 mm) column thermostated at 40 °C. The mobile phase flow rate was 0.4 mL∙min^−1^, and elution was performed using 0.1% (*v*/*v*) formic acid in water (solvent A) and can/MeOH (1:1; *v*/*v*) (solvent B) in gradient mode: 10% B to 100% B in 20 min. The UV signal was registered at 254 nm. All mass-spectrometric scan data were recorded in negative ionization scan mode. The nebulizer pressure, nitrogen flow rate, drying gas temperature, drying gas flow rate, and sheath gas temperature were 45 psi, 5 L∙min^−1^, 300 °C, 11 L∙min^−1^, and 250 °C, respectively. The capillary voltage was 3.5 kV, and the fragmentation voltage was 200 V. The collision cell radio frequency voltage was deactivated, the first quadrupole was in total transmission ion mode, and the second quadrupole was scanning, resulting in the triple quadrupole mass spectrometer being in scan mode. In this mode, the fragmentor voltage regulates the rate at which ions pass through a medium pressure zone and are fragmented by collisions with nitrogen molecules.

### 2.5. Theoretical Calculations

The stationary point structures of all systems investigated were obtained by applying the Density Functional Theory (DFT) method with the B3LYP [28,29] functional and the 6-311++G(d,p) [30,31] basis set for all atoms. The choice of the B3LYP/6-311++G(d,p) theoretical treatment was dictated by the trustworthiness and cost effectiveness of this approach, as many earlier reports [32,33,34,35,36] confirmed its usefulness and reliability in studying molecular structures (with the average absolute errors of 0.013 Å, 0.62°, and 0.35° in reproducing bond lengths, valence angles, and dihedral angles, respectively) and reaction mechanisms (with the average absolute error of 0.98 and 2.20 kcal/mol in reproducing zero-point energies and atomization energies, respectively) [37].

The harmonic vibrational frequencies characterizing the stationary points were evaluated (without scaling) at the same level of theory to ensure that all obtained structures corresponded to either true minima or first-order saddle points (i.e., transition states) on the potential energy surface. The intrinsic reaction coordinate procedure (IRC) was employed to confirm the minima for each transition structure at the B3LYP/6-311++G(d,p) theory level. The partial atomic charges were fitted to the electrostatic potential according to the Merz–Singh–Kollman scheme [38]. All calculations were carried out using the GAUSSIAN16 (Rev.B.01) package [39].

## 3. Results

### 3.1. HPLC-ESI(-)-MS Study

**Figure 2:**
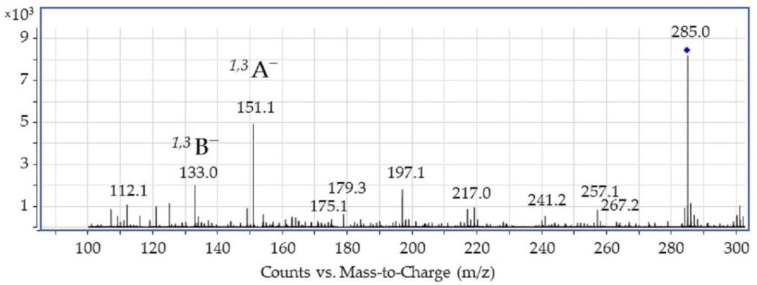
ESI-MS spectrum of luteolin acquired in negative ion mode.

Luteolin was isolated from weld raw plant material according to hydrofluoric acid procedure (see the preceding section) and then characterized by high-performance liquid chromatography–mass spectrometry (HPLC-ESI-MS) using atmospheric pressure electrospray ionization in negative mode (see Figure 2 and Table 1). The mass spectrum of luteolin showed the presence of characteristic ions at *m*/*z*: 267, 257, and 241 formed after neutral losses of H_2_O, CO, and 2CO_2_, which may be attributed to the **C** ring [15,22]. Another small neutral loss corresponds to the C_3_O_2_ (−68 Da) cleavages (*m*/*z* at 217), and this fragment ion undergoes further C_2_H_2_O loss, leading to the fragment *m*/*z* at 175 (both fragmentations occur within the **A** ring of the luteolin molecule).

The most intensive fragment ions corresponding to retro-Diels–Alder (RDA) fragmentation of luteolin gave rise to two species, namely the ^1,3^B^−^ ion at *m*/*z* 133 and the ^1,3^A^−^ ion at *m*/*z* 151. As revealed by the ESI-MS spectrum shown in Figure 2, the ^1,3^A^−^ fragment is the main fragment ion of luteolin. It should also be noted that a direct cleavage of the bond connecting the **B** and **C** rings which would result in the appearance of the [M-**B**]^−^ fragment was not detected, although this type of cleavage can be observed for some flavonols (i.e., flavonoids with a 3-hydroxyflavone backbone) containing a hydroxyl group in the **C** ring, such as quercetin (3,3′,4′,5,7-Pentahydroxyflavone) or fisetin (7,3′,4′-flavon-3-ol) [15,21,22]. We refer to this issue (i.e., the absence of the [M-**B**]^−^ fragment ion) in the following sections.

### 3.2. Isomeric Structures Resulting from Deprotonation of Neutral Luteolin

**Figure 3:**
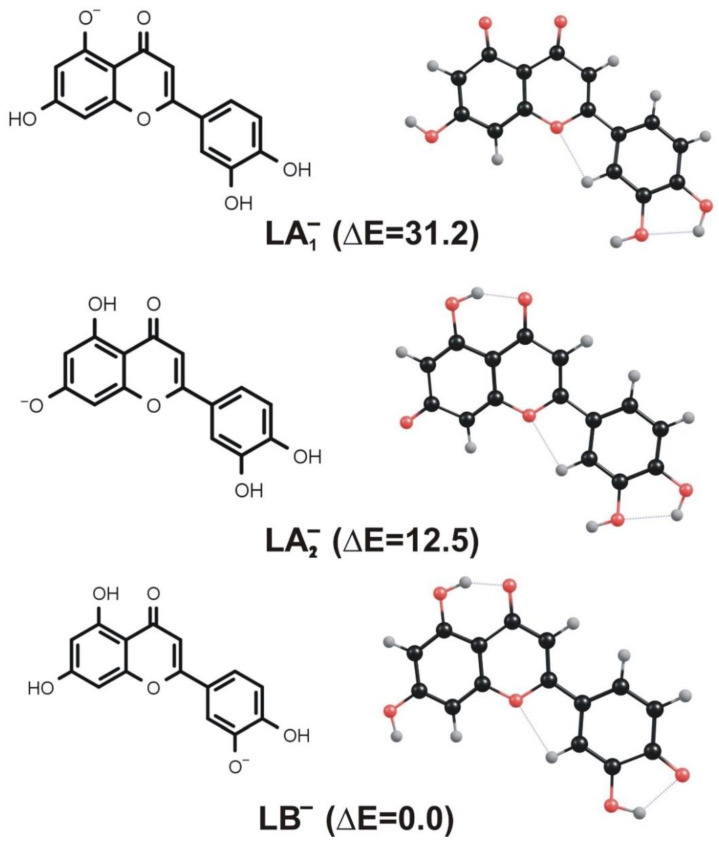
Equilibrium structures of LA_1_^−^, LA_2_^−^, and LB^−^ anions resulting from deprotonation of neutral luteolin. Relative energies (ΔE) of these isomers are given in kcal/mol.

Since the neutral luteolin molecule (L) depicted in Figure 1 contains four deprotonation sites (represented by hydroxyl groups), the proton detachment may lead to four different isomeric negatively charged structures. Two of those anionic isomers result from the deprotonation of the OH groups connected to the **A** ring (we label them LA_1_^−^ and LA_2_^−^ in Figure 3), whereas the remaining two isomers are formed when either of the hydroxyl groups attached to the **B** ring is deprotonated. However, the latter pair of isomers are nearly isoenergetic and may easily evolve into each other via the proton transfer between two neighboring OH groups (we verified that the kinetic barrier for such a proton transfer is rather small and equal to 7 kcal/mol). Therefore, in our further investigation (concerning the systems formed by deprotonation of the **B** ring substituents), we decided to consider only one negatively charged isomer resulting from the deprotonation of the OH group connected to the **B** ring (see the structure labeled LB^−^ in Figure 3).

Even though the anionic LA_1_^−^, LA_2_^−^, and LB^−^ isomers considered differ with one another only by the position of deprotonated hydroxyl group, their total electronic energies vary significantly. Namely, the LB^−^ anion corresponds to the lowest energy structure, while the relative energies of LA_1_^−^ and LA_2_^−^ isomers were found to be larger by 31.2 and 12.5 kcal/mol, respectively (see Figure 3). If one considered the possible existence of these isomeric structures in the equilibrium conditions, such relative energies would clearly indicate that the mixture should be dominated by the LB^−^ species, whereas the presence of LA_1_^−^ and LA_2_^−^ structures in the bulk should be considered unlikely. However, the deprotonation of luteolin caused by the negative ion electrospray ionization technique likely leads to the formation of all isomeric structures depicted in Figure 3, which are then the subjects of the fragmentation processes that follow. Therefore, a thorough study of deprotonated luteolin fragmentation has to cover the paths which involve all LA_1_^−^, LA_2_^−^, and LB^−^ isomers as starting substrates, more so because it is not experimentally feasible to recognize the isomer type while performing ESI-MS. In the following sections, we present the energy profiles that correspond to the fragmentation channels resulting in the formation of both ^1,3^A^−^ (at *m*/*z* 151) and ^1,3^B^−^ (at *m*/*z* 133) anions observed in the ESI-MS experiment.

### 3.3. Fragmentation Paths for LA_1_^−^ Isomer (Leading to ^1,3^A^−^ Product at m/z 151)

The 1,3-retrocyclization cleavage of the LA_1_^−^ anion involves the rupture of the C-O bond (labeled 1 in Figure 1) and the rupture of the C-C bond (labeled 3 in Figure 1) in the **C** ring. According to our theoretical predictions, this process may proceed according to either a concerted or stepwise mechanism.

**Figure 4:**
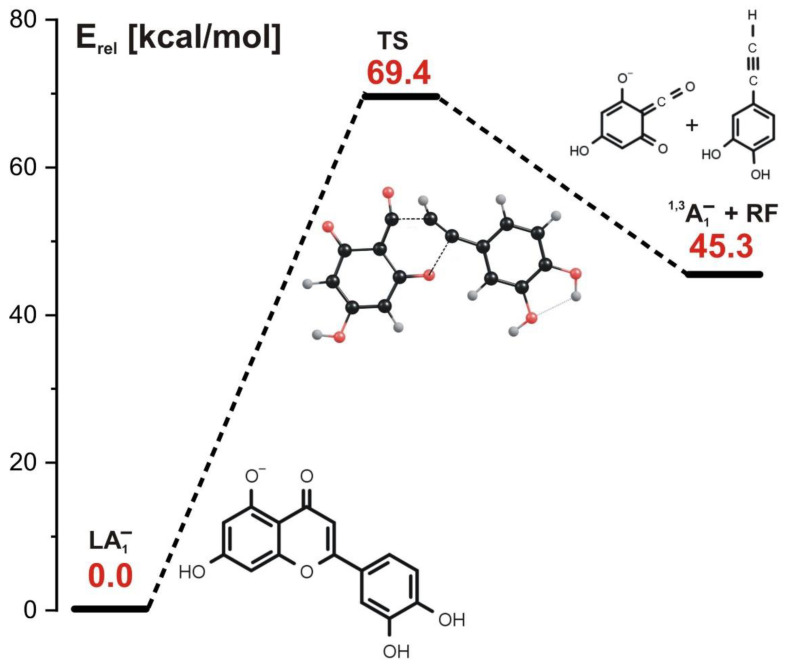
Energy profile for the 1,3-retrocyclization of LA_1_^−^ proceeding according to the concerted mechanism and resulting in the formation of ^1,3^A_1_^−^ negatively charged product. The energies of the transition state (TS) and the separated products (^1,3^A_1_^−^ anion and the remaining neutral fragment RF) are given (in kcal/mol) as the relative values (E_rel_) with respect to the energy of the LA_1_^−^ substrate (taken as zero).

In the concerted mechanism, the simultaneous cleavage of two bonds in the **C** ring occurs. As indicated in the energy profile for this path (depicted in Figure 4), such a process requires a single kinetic barrier whose height approaches 70 kcal/mol to be overcome. The structure of the transition state corresponding to this barrier (labeled TS in Figure 4) reveals two elongated bonds (i.e., C-O and C-C) which are simultaneously ruptured along that reaction path. As a result, the ^1,3^A_1_^−^ negatively charged product and the remaining neutral fragment (labeled RF) are formed; the former is observed as ^1,3^A^−^ at *m*/*z* 151 in the ESI-MS experiment, whereas the latter is not charged and not observed as such.

**Figure 5:**
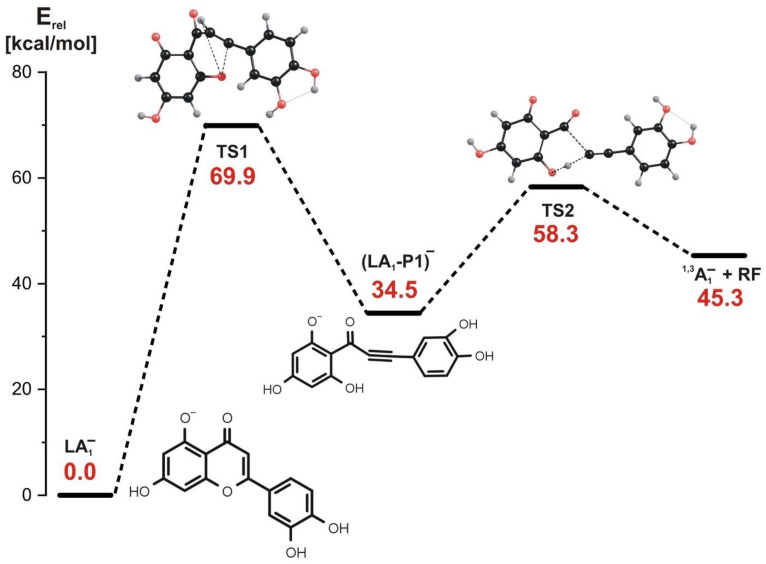
Energy profile for the 1,3-retrocyclization of LA_1_^−^ proceeding according to the stepwise mechanism and resulting in the formation of ^1,3^A_1_^−^ negatively charged product. The energies of the transition states (TS1 and TS2), intermediate product ((LA_1_-P1)^−^), and the separated products (^1,3^A_1_^−^ anion and the remaining neutral fragment RF) are given (in kcal/mol) as the relative values (E_rel_) with respect to the energy of the LA_1_^−^ substrate (taken as zero).

In the stepwise mechanism whose energy profile is shown in Figure 5, two kinetic barriers have to be surmounted to achieve the fragmentation products. The first reaction step involves the rupture of the C-O bond in the **C** ring and a simultaneous H transfer from carbon atom to oxygen atom (see the structure of the corresponding transition state labeled TS1 in Figure 5). This step requires the kinetic barrier of nearly 70 kcal/mol to be overcome and results in the formation of the intermediate product labeled (LA_1_-P1)^−^, whose relative energy is larger by 34.4 kcal/mol than that of the LA_1_^−^ substrate, as shown in Figure 5. The examination of the bond lengths in the (LA_1_-P1)^−^ structure reveals that it contains a triple C≡C bond connecting the **C** ring and the remaining part of the molecule.

In the second reaction step, the (LA_1_-P1)^−^ intermediate product undergoes the rupture of the C-C bond and a simultaneous H transfer from the oxygen atom back to the carbon atom. The height of the kinetic barrier for this process was estimated to be 23.8 kcal/mol (see also the corresponding transition state structure labeled TS2 in Figure 5). As in the concerted mechanism described in the preceding paragraph, the products of the LA_1_^−^ fragmentation proceeding along the stepwise mechanism involve the ^1,3^A_1_^−^ negatively charged product (observed as ^1,3^A^−^ at *m*/*z* 151 in the ESI-MS) and the remaining neutral fragment (labeled RF in Figure 5). Hence, we conclude that both described mechanisms (i.e., concerted and stepwise) are likely operative in the fragmentation of LA_1_^−^ and they are equally plausible due to the similar heights of the largest kinetic barriers estimated for these paths.

### 3.4. Fragmentation Paths for LA_2_^−^ Isomer (Leading to ^1,3^A^−^ Product at m/z 151)

As was the case for the LA_1_^−^ anion (see the preceding section), the 1,3-retrocyclization cleavage of the LA_2_^−^ anion also involves the rupture of two bonds (C-O and C-C) in the **C** ring. Again, our calculations indicate that such a process may proceed according to either the concerted or stepwise mechanism.

**Figure 6:**
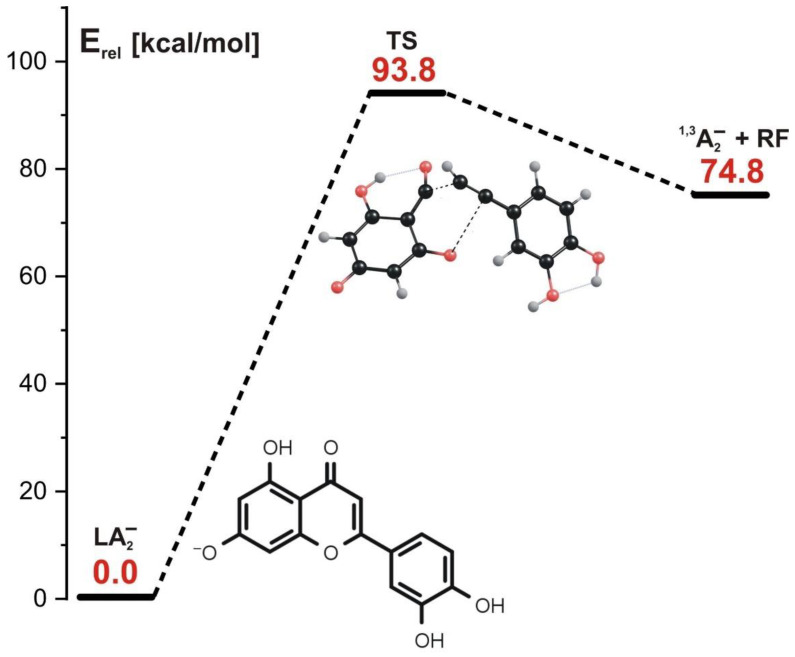
Energy profile for the 1,3-retrocyclization of LA_2_^−^ proceeding according to the concerted mechanism and resulting in the formation of ^1,3^A_2_^−^ negatively charged product. The energies of the transition state (TS) and the separated products (^1,3^A_2_^−^ anion and the remaining neutral fragment RF) are given (in kcal/mol) as the relative values (E_rel_) with respect to the energy of the LA_2_^−^ substrate (taken as zero).

The transition state structure found for the concerted mechanism (see the structure labeled TS in Figure 6, where the energy profile for this reaction is also depicted) reveals that both C-O and C-C bonds are substantially elongated. The formation of the final products (^1,3^A_2_^−^ and the remaining neutral fragment labeled RF in Figure 6) requires the kinetic barrier whose height we estimated to be 93.8 kcal/mol to be overcome. Interestingly, this barrier is larger by ca. 24 kcal/mol than that predicted for the concerted mechanism involving the LA_1_^−^ anion as a substrate. We believe that such a difference might be caused by the larger stability of LA_2_^−^ in comparison to the LA_1_^−^ isomer, as the relative energy of the latter exceeds that of the former by nearly 19 kcal/mol, as shown in Figure 3.

**Figure 7:**
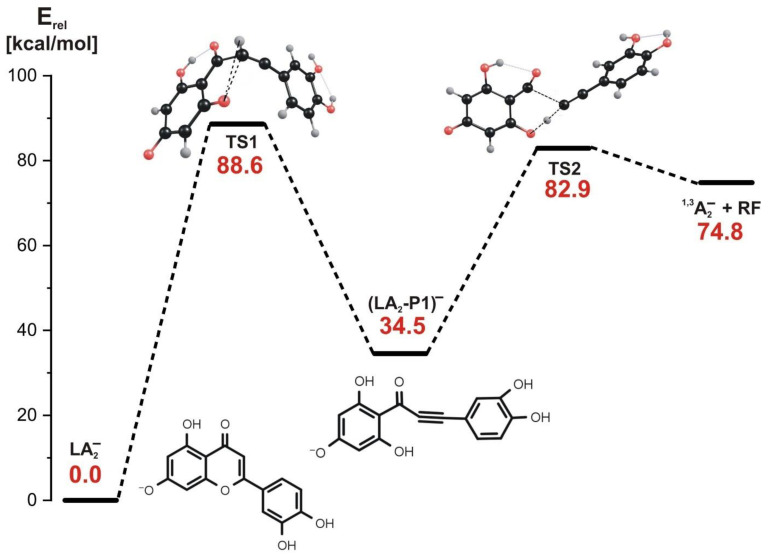
Energy profile for the 1,3-retrocyclization of LA_2_^−^ proceeding according to the stepwise mechanism and resulting in the formation of ^1,3^A_2_^−^ negatively charged product. The energies of the transition states (TS1 and TS2), intermediate product ((LA_2_-P1)^−^), and the separated products (^1,3^A_2_^−^ anion and the remaining neutral fragment RF) are given (in kcal/mol) as the relative values (E_rel_) with respect to the energy of the LA_2_^−^ substrate (taken as zero).

As far as the stepwise mechanism is concerned, our calculations indicate that the first reaction step involves both the rupture of the C-O bond in the **C** ring and a simultaneous hydrogen transfer from carbon to oxygen, as shown in the structure labeled TS1 in Figure 7, where the transition state structure for this process is depicted.

This first step requires the kinetic barrier whose height approaches 90 kcal/mol to be surmounted, and it leads to the intermediate product (labeled (LA_2_-P1)^−^ in Figure 7) in which the initial **C** ring is opened, and the **B** ring becomes connected with the remaining molecular framework via a triple C≡C bond. In the second reaction step, the energy barrier of 48.4 kcal/mol has to be overcome, as shown in the energy profile shown in Figure 7. As indicated by the transition state structure (labeled TS2 in Figure 7) whose relevance was confirmed by the IRC calculations, the C-C bond is ruptured in this reaction step. In addition, this process involves the hydrogen transfer from the oxygen atom back to the carbon atom. Hence, the final products of the reaction proceeding via the stepwise mechanism are the same as those achieved in the concerted process involving the LA_2_^−^ substrate and consist of the ^1,3^A_2_^−^ anion and the non-charged (neutral) molecular fragment (labeled RF).

Since the kinetic barriers predicted for the 1,3-retrocyclization cleavage of the LA_2_^−^ anion proceeding either along the concerted or the stepwise path are similar (ca. 89–94 kcal/mol), one may conclude that both mechanisms are operative and seem almost equally plausible in the ESI-MS experiment.

It should be noted that although the fragmentation involving the LA_2_^−^ anion as a substrate leads to the ^1,3^A_2_^−^ anion, whereas the fragmentation of the LA_1_^−^ anion (described in the preceding section) produces a structurally different ^1,3^A_1_^−^ anion, both these processes (each of which could proceed along either concerted or stepwise path) result in the formation of negatively charged species which are identified as ^1,3^A^−^ anions at *m*/*z* 151 in the ESI-MS apparatus, as ^1,3^A_1_^−^ and ^1,3^A_2_^−^ systems are experimentally indistinguishable in such measurements.

### 3.5. Fragmentation Paths for LB^−^ Isomer (Leading to ^1,3^B^−^ Product at m/z 133)

Unlike the 1,3-retrocyclization cleavage of either the LA_1_^−^ or LA_2_^−^ anion, the analogous reaction involving the LB^−^ anion as a substrate can proceed according to the stepwise mechanism alone. In other words, we verified that the concerted route is not possible for the fragmentation process of LB^−^ yielding ^1,3^B^−^, as the transition state structure for a simultaneous rupture of two bonds (i.e., C-O and C-C) in the **C** ring does not exist. We confirmed the inexistence of such a transition state by performing several unsuccessful searches for the appropriate saddle point structure using various techniques (including the Berny algorithm, STQN/QST2, and STQN/QST3) designed to locate first-order saddle points. Hence, here, we present the energy profile of the stepwise reaction, which is the only possible path leading to the formation of the ^1,3^B^−^ anions observed in the ESI-MS experiment at *m*/*z* 133.

**Figure 8:**
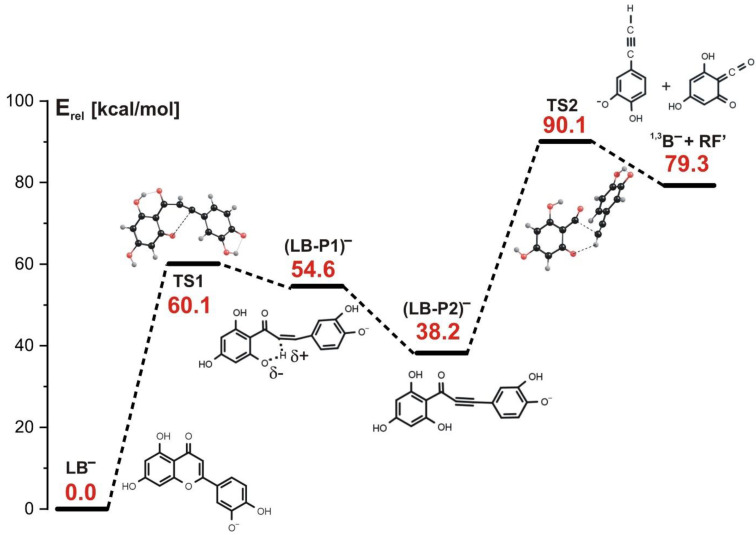
Energy profile for the 1,3-retrocyclization of LB^−^ proceeding according to the stepwise mechanism and resulting in the formation of ^1,3^B^−^ negatively charged product. The energies of the transition states (TS1 and TS2), intermediate products ((LB-P1)^−^ and (LB-P2)^−^), and the separated final products (^1,3^B^−^ anion and the remaining neutral fragment RF’) are given (in kcal/mol) as the relative values (E_rel_) with respect to the energy of the LB^−^ substrate (taken as zero).

Similarly to the initial step of the stepwise reactions involving either the LA_1_^−^ or LA_2_^−^ anion as a substrate (see the preceding sections), the 1,3-retrocyclization cleavage of LB^−^ begins with the rupture of the carbon–oxygen bond in the **C** ring; however, this bond breakage is not accompanied by H transfer (as it was the case for both LA_1_^−^ or LA_2_^−^ systems). This first reaction step requires the kinetic barrier of 60.1 kcal/mol to be surmounted and proceeds via the transition state structure labeled TS1 in Figure 8.

The equilibrium structure of the intermediate product (labeled (LB-P1)^−^ in Figure 8) formed after passing this first barrier contains a significantly elongated C-H bond (r(C-H) = 1.624 Å) with positive partial charge on the H atom involved (+0.4|e|). Additionally, the neighboring oxygen atom holds a substantial negative partial charge (−0.7|e|) which results in the formation of the C-H^δ+^⋯O^δ−^ fragment. This in turn gives rise to the barrier-free proton transfer that follows. Namely, our calculations indicate that in the second reaction step, the (LB-P1)^−^ intermediate whose structure corresponds to a very shallow minimum undergoes a barrierless evolution involving the proton transfer from carbon to oxygen, which leads to another intermediate product (LB-P2)^−^ whose energy is smaller by 16.4 kcal/mol than the energy of (LB-P1)^−^, as shown in Figure 8. In consequence, a triple C≡C bond and a hydroxyl group are formed, as shown in the structural formula of (LB-P2)^−^. Next, another kinetic barrier (whose height was calculated to be 51.9 kcal/mol) has to be overcome to generate the final reaction products, namely the ^1,3^B^−^ anion and the remaining neutral fragment (RF’), as shown in Figure 8. The structure of the transition state corresponding to that third reaction step (labeled TS2 and depicted in Figure 8) indicates that the C-C bond is ruptured (likely heterolytically) and the proton is transferred from the oxygen atom back to the carbon atom. Such a sequence of reaction steps enables the formation of the ^1,3^B^−^ anion, which is observed in the ESI-MS experiment at *m*/*z* 133.

### 3.6. Alternative Fragmentation Paths for LA_1_^−^, LA_2_^−^, and LB^−^ Isomers

**Figure 9:**
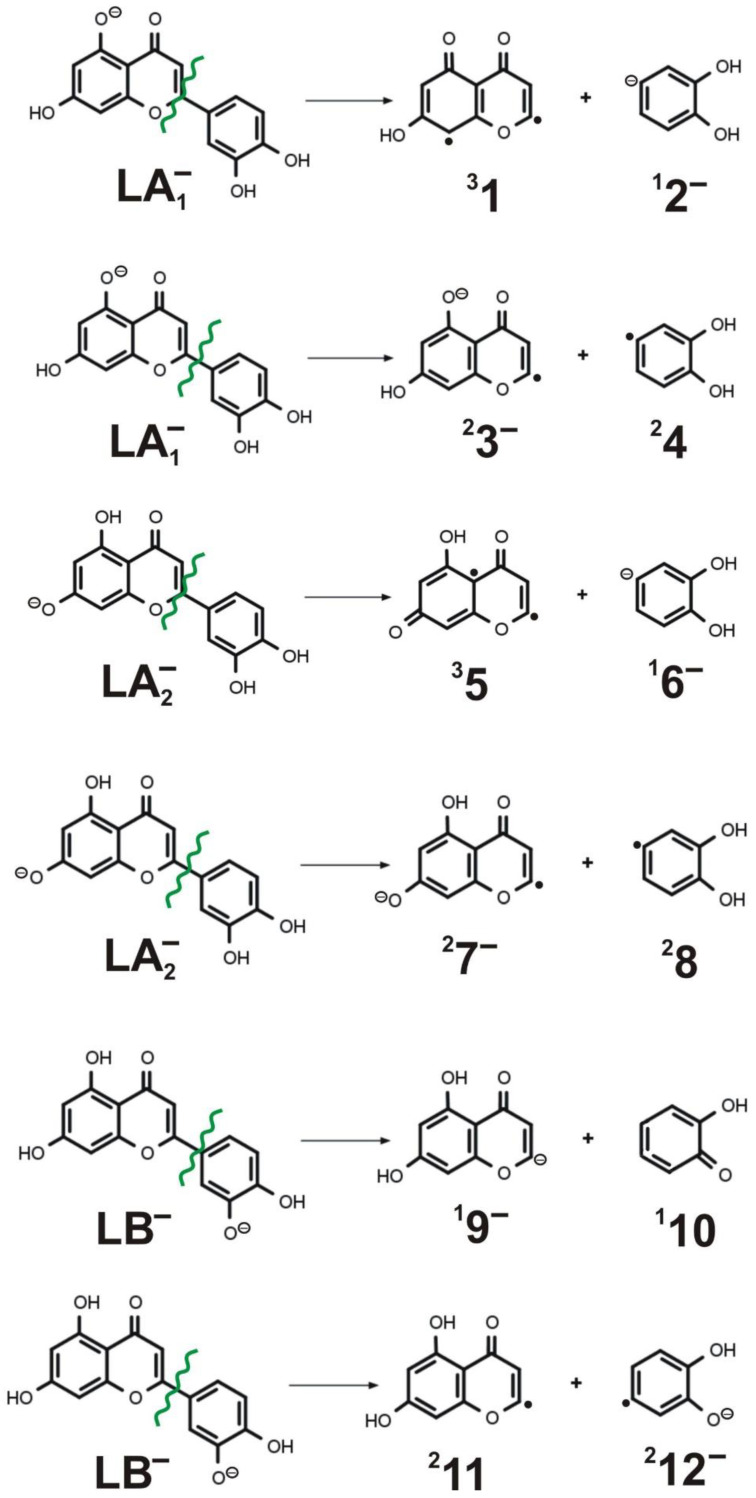
The fragmentations of LA_1_^−^, LA_2_^−^, and LB^−^ caused by either heterolytic or homolytic cleavage of the C-C bond connecting **C** and **B** rings. Spin multiplicity and the charge of the **1**–**12** products are indicated in the left and right superscript, respectively.

Having discussed the concerted and stepwise mechanisms of the 1,3-retrocyclization cleavage of LA_1_^−^, LA_2_^−^, and LB^−^ anions leading to the ^1,3^A^−^ and ^1,3^B^−^ negatively charged fragments observed in the ESI-MS experiment, we now move on to describing an alternative path of ionized luteolin fragmentation. First, let us explain that it was not our goal to consider all possible fragmentation routes other than 1,3-retrocyclization cleavage (such as 1,2-, 1,4-, or 0,4-retrocyclization cleavages). Instead, we decided to choose a fragmentation path which is not operative in the ESI-MS experiment. We did so hoping to make the comparison of the barrier heights between such a non-operative mechanism and the operative mechanisms studied in this contribution. Therefore, knowing that the single C-C bond connecting the **B** and **C** rings in the negatively ionized (i.e., deprotonated) luteolin (see Figure 1) is not prone to any detectable cleavage during the ESI-MS measurements, we chose that particular bond for our investigation. In particular, we considered both homolytic and heterolytic C-C cleavages (see Figure 9), although in the latter case, we did not include the heterolytic bond ruptures that would lead to two oppositely charged ionic fragments (i.e., dianion and cation pairs), as the formation of such ionic pairs is highly unlikely.

As shown in Figure 9, the homolytic C-C bond cleavage always produces two radicals (a radical anion and a neutral radical) whose ground electronic states are doublets (i.e., spin multiplicity is equal to 2), as shown in the fragmentations numbered (2), (4), and (6) in Table 1, where the reaction energies for all these processes are collected. On the other hand, the heterolytic cleavages may lead to various pairs of fragments, one of which is always a closed-shell singlet anion (^1^**2^−^** in (1), ^1^**6^−^** in (3), and ^1^**9^−^** in (5)) and the other one is either a biradical neutral in the triplet state (^3^**1** in (1) and ^3^**5** in (3)) or a closed-shell singlet neutral (^1^**10** in (5)). Since we suspected that the open-shell singlet states of the neutral systems **1** and **5** (which are also biradical species) could be competitive with their corresponding triplet states, we verified that it is not case (as these open-shell singlet states are close in energy yet less energetically stable than the triplet states).

As one might have anticipated, the reaction energies corresponding to the homolytic cleavages are smaller than those obtained for the heterolytic ones, as shown in Table 2. Namely, the reaction energies predicted for the homolytic fragmentations (2), (4), and (6) span the range of 131–134 kcal/mol, whereas those calculated for the heterolytic ruptures span the 137–166 kcal/mol range (with the smallest one of 136.7 kcal/mol corresponding to the reaction (5) yielding two closed-shell singlet species). Nevertheless, all the reaction energies obtained for the cleavages of the C-C bond connecting **C** and **B** rings in LA_1_^−^, LA_2_^−^ and LB^−^ are substantially larger (by 61–85 kcal/mol for LA_1_^−^, 40–72 kcal/mol for LA_2_^−^, and by 44–47 kcal/mol for LB^−^) than the activation energies found for the 1,3-retrocyclizations, as the results described in the preceding sections and Table 2 affirm. Therefore, we conclude that the products of (1)–(6) fragmentations are not observed while performing ESI-MS measurements due to much larger energies that would have to be applied to generate such cleavages.

## 4. Summary and Conclusions

On the basis of both (i) the B3LYP/6-311++G(d,p) calculations performed for deprotonated luteolin undergoing retro-Diels–Alder fragmentation and (ii) the results of high-performance liquid chromatography coupled to electrospray ionization tandem mass spectrometry (LC-(ESI-)-MS), we arrive at the following conclusions:

(i) Two of the most intensive spectral features observed in the ESI-MS spectrum of luteolin (acquired in negative ion mode) correspond to the ^1,3^A^−^ ion at *m*/*z* 151 and the ^1,3^B^−^ ion at *m*/*z* 133 (with the former being the main fragment ion observed).
(ii) The presence of two fragment ions (i.e., ^1,3^A^−^ and ^1,3^B^−^) in the ESI-MS spectrum and the absence of the signal corresponding to the [M-**B**]^−^ fragment ion (which would appear if the bond connecting luteolin’s **B** and **C** rings were ruptured) indicates that the luteolin molecule undergoes the 1,3-retrocyclization process, whereas a direct cleavage of a single C-C bond representing the linkage between the aromatic rings (observed for some flavonols) does not occur in this case.
(iii) Theoretical studies indicate that the 1,3-retrocyclization of deprotonated luteolin may proceed according to either the concerted or stepwise mechanism (when one of the hydroxyl groups connected to the **A** ring is initially deprotonated) or according to the stepwise mechanism only (when the initial deprotonation is related to one of the OH groups connected to the **B** ring).
(iv) (In the concerted mechanism involving LA_1_^−^ or LA_2_^−^ ionized luteolin isomer as a starting structure, the simultaneous cleavage of two bonds (C-O and C-C) in the **C** ring occurs. Such a process requires a single kinetic barrier whose height is equal to 69 kcal/mol for LA_1_^−^ and 94 kcal/mol for LA_2_^−^ to be overcome and results in the formation of ^1,3^A_1_^−^ or ^1,3^A_2_^−^ anions which are experimentally indistinguishable (as they both appear as the fragment ion at *m*/*z* 151 in ESI-MS spectrum).
(v) In the stepwise mechanism involving LA_1_^−^ or LA_2_^−^ as a starting structure, two kinetic barriers have to be surmounted: the first barrier (70–89 kcal/mol, depending on the isomer) is related to the reaction step involving the rupture of the C-O bond in the **C** ring and a simultaneous H transfer from C to O, and the second barrier (24–48 kcal/mol, depending on the isomer) is related to the next step involving the rupture of the C-C bond and a simultaneous H transfer from the O atom back to the C atom. Regardless of the starting structure considered (LA_1_^−^ or LA_2_^−^), the stepwise fragmentation path leads to the formation of the final product appearing as ^1,3^A^−^ ion at *m*/*z* 151 in ESI-MS spectrum.
(vi) The fragmentation involving the LB^−^ isomer as a starting reagent proceeds according to the stepwise mechanism (which is the only operative mechanism for this isomer) and involves three steps: the first step related to the C-O bond cleavage (with the barrier of 60 kcal/mol), the barrierless proton transfer from C to O as the second step, and the final step (requiring the activation energy of 52 kcal/mol) related to both the C-C bond rupture and the proton transfer from O back to C. This reaction path leads to the formation of the ^1,3^B^−^ anion observed in the ESI-MS experiment at *m*/*z* 133.
(vii) The absence of the spectral feature corresponding to the [M-**B**]^−^ fragment in the ESI-MS spectrum is likely caused by the fact that such a fragment ion formation would require much higher energy barriers (131–166 kcal/mol) related to the rupture of the C-C bond connecting luteolin’s **B** and **C** rings to be surmounted.

## Figures and Tables

**Table 1:** ESI-MS product ions obtained for luteolin.

MS (*m*/*z*)	Fragment Ions
285	[M-H]**^−^**
267	[M-H-H_2_O]**^−^**
257	[M-H-CO]**^−^**
241	[M-H-CO_2_]**^−^**
217	[M-H-C_3_O_2_]**^−^**
197	[M-H-2CO_2_]**^−^**
175	[M-H-C_3_O_2_-C_2_H_2_O]**^−^**
151	^1,3^A**^−^**
133	^1,3^B**^−^**

**Table 2:** Reaction energies (in kcal/mol) corresponding to the homolytic and heterolytic fragmentations depicted in Figure 9. The results are obtained by employing the B3LYP method and the 6-311++G(d,p) basis set.

Process No.	Fragmentation Reaction	Cleavage Type	Reaction Energy
(1)	LA_1_^−^ → ^3^**1** + ^1^**2**^−^	heterolytic	156.5
(2)	LA_1_^−^ → ^2^**3**^−^ + ^2^**4**	homolytic	131.3
(3)	LA_2_^−^ → ^3^**5** + ^1^**6**^−^	heterolytic	165.6
(4)	LA_2_^−^ → ^2^**7**^−^ + ^2^**8**	homolytic	134.2
(5)	LB^−^ → ^1^**9**^−^ + ^1^**10**	heterolytic	136.7
(6)	LB^−^ → ^2^**11** + ^2^**12**^−^	homolytic	134.3
